# A System of Surgery

**Published:** 1847-10-01

**Authors:** 


### A System of Surgery. By *J. M. Chelius*. Translated from the German, with additional Notes and Observations. By *John F. South*. Two vols., 8vo., pp. 1823. Analyt. Index, clxx. Renshaw, 1847.

Although we have noticed this work more than once during its progress of publication in parts (which have been issued with exemplary regularity), we do not deem it right to withhold our expression of congratulation from the translator upon the satisfactory conclusion of his really laborious task. It will supply a want hitherto felt of a due exposition of the present condition of German Surgery in this country; for, as Mr. South observes, no standard work relating to this has appeared in English since the publication of Heister's book a century since. In his preface, and incidentally during the work, he speaks somewhat too disparagingly of French Surgery, "with its showy, but somewhat too hazardous operations;" but we are of opinion that not only are English students and practitioners more familiar with this last than with German surgery in consequence of the greater diffusion of a knowledge of the French language, and the greater intercourse between Paris and London, but also because of its intrinsic superiority to that of any continental nation whatever. And in proof of this, we need not go farther than the book now before us; for while a translation of a work from the pen of a French surgeon and teacher occupying a position similar to that of Chelius might easily and advantageously be presented to the British public in *puris naturalibus*, we do not hesitate to say, in regard to the present one, that had it not been for the valuable and numberless additions of the translator, in the shape of amplifications, illustrations, protests, corrections, and emendations, it would have infallibly fallen still-born from the press. Shorn of these, it is incomparably inferior, and in some respects half-a-century behind, our own manuals. Mr. South has, however, so hedged in his author's text by notes upon notes derived from his own experience or reading, that, however uncomplimentary such a procedure may be to his author, and however undesirable such a precedent is for general imitation, he has succeeded in converting the original meagre "Handbuch der Chirurgie" into a tolerable complete System of Surgery, which both students and practitioners will find very useful as a work of reference, and the employment of which is facilitated by the addition of an admirable index, of dimensions seldom seen now-a-days, amply compensating for the defective arrangement of the matter of the work.

We may now present our readers with a few specimens of the contents.

*Of the Elementary Proceedings of Surgical Operations.*—Considering the economy of space aimed at in the "Handbuch," (a principle utterly repudiated by the translator however,) one cannot help being somewhat amused at some of the unnecessary and Germanic details entered into, concisely enough it is true, in this section.

"There is scarcely a surgical operation which can be fully perfected on a diseased body by one single, simple act. All rather consist of several manœuvres following, according to determined rules, and distinguished by the name of *Steps of the Operation*. One of these is the special object of the operation, and the others must necessarily precede or follow, to effect this object, and bring about the restoration of the patient. The object of the operation is always the same, but the manner and way of attaining it may be very different, and this difference may consist either in the difference of the several steps of the operation, or of the entire way by which the attainment of the object of the proposed operation may be effected. Hence arises the distinction between *Operative Proceedings* and *Methods of Operating*.

"The *Method of Operating* is the compass of the regulated modes of proceeding, by which the object of an operation in any peculiar way is attained. In the various methods of operating, therefore, not merely are different parts cut through and in very different directions, but the practice of the methods of operating is so peculiar, that the one method does not exclude the other. Upon the choice of the method of operating depends, for the most part, the successful or unsuccessful result of the operation, just as upon the choice of the operative proceedings rests the facility of its execution. The choice of the mode of proceeding is therefore of little consequence, and depends commonly on the operator himself. Hence, also, the variety of opinions as to the mode of proceeding in general, is greater than upon that of the method.

"In deciding upon the preference of the various methods of operation, the following circumstances must be attended to. *First.* The least important organs must be injured, consequently the loss or destruction of organic parts caused by the operation, the pain and the traumatic reaction depending thereon is least. *Second.* The better method must always be most fitting for the greater number of cases. *Third.* This must consist in the manœuvres, which do not make the operator dependent upon accidental circumstances, but which rest completely on the will of the operator. Hereon and upon the nature of the parts to be wounded, are founded safety and facility in the execution of the method of operating. *Fourth.* The quickest cure which can be effected by the operation." Vol. II., p. 852.

The following circumstances require attention for the securing a probability of a successful result. 1. The disease to be removed should not be so connected with any general ailment, that this may act on it as a cause to keep it up continually. This would only be to remove the product of general disease, leaving the producing cause. The best results however follow when the general disease terminates in a local disease capable of removal. 2. The patient's weakness or sensibility should not be so excessive as to endanger life by the operation. 3. The local disease must not, by its long-continuance or other relations to the constitution, have acquired the rank of a secreting organ, or have removed any previously existing disease, or have checked it in its earlier development. Cases may occur, however, where, in spite of a cure not being anticipated from an operation, this may be employed as a means of mitigating suffering or prolonging existence.

It respect to the patient's condition, in those who can bear pain quietly and patiently, are operations least dangerous. Those, too, who have been long accustomed to, and enfeebled by, painful disease, through the moral influence of the desire for the operation, and the less amount of traumatic reaction, bear them best. Persons of plethoric temperament, full of apparent health, or such as are very stout, of tall and strong make, bear them ill. Nervous persons may be placed in two categories—those "who are very sensitive and excitable, and those who, on the slightest cause, drop into moral despondency and nervous stupidity"—the former, although much affected by the pain, soon rally under the influence of hope and encouragement, which is not the case with the latter. The young bear operations better than the old, although in advanced age operations are not contra-indicated, and even often succeed better, in consequence of the less amount of traumatic reaction. In gouty subjects they are dangerous, and in the scrofulous the removal of a diseased part may be followed by the active development of scrofula in some internal organ. The nature of the preparation the patient is submitted to will depend upon the prevalence of any of the above circumstances—according to which we may endeavour to counteract general disease, raise his powers, depress augmented sensibility, &c. Robust and full-blooded persons should be kept some time on spare diet, and bleeding resorted to if this seems indicated. Great sensibility may be somewhat suppressed by opiates given before, and soon after, the operation.

As a general rule, the translator is, however, and we think properly, opposed to the use of *opium* or other sedative, owing to the difficulty of distinguishing between the effect of the medicine and the symptoms arising from constitutional excitement; and he believes that, even in the case of persons habituated to the use of opium subjected to operations, the drug may be diminished or withheld sooner than is usually believed. The mere absence of the torturing pain they have been so long accustomed to seek its aid against sufficing to allow of sleep. If, however, the patient do not sleep, as the obtaining this is paramount, he does not withhold an opiate, whether the individual has been in the habit of taking it or not—while free livers may even require its repetition two or three times a day during the whole course of their case, keeping the bowels open at the same time with an occasional dose of three or four grains of calomel, which acts upon, without irritating the canal. Mr. South adds—

"Another very important point in the treatment of operations is the use of *porter, wine,* or spirituous *liquors.* Even where the patient has been prudent and temperate, it is occasionally necessary that one or other of these should be given soon after an operation. But for persons who have been accustomed to take large quantities of porter or spirits, or both, and who, in consequence of severe accidents, are subjected to the amputation of a limb, or who have severe lacerations, which, however, do not require operation, it is absolutely necessary for their safety that the stimulant should not only be not entirely withdrawn, but even somewhat very near the quantity they have been accustomed to, must be allowed, or they either sink at once, are attacked with erysipelas, or are violently affected with *delirium tremens,* in which condition they speedily die. The quantity taken may often seem enormous under the circumstances; three or four glasses of gin or brandy, and as much or more wine, and sometimes porter besides, in the course of the 24 hours, is by no means an unfrequent allowance; and I have just the recollection of one of the younger Cline's patients, a porter in the Royal Exchange, who required a pint of brandy daily after having suffered amputation of his leg after an accident. This man was saved by this treatment, and lived many years after, doubtless following the same free course of living which had required treatment, at that period thought exceedingly bold and almost marvellous in its result, although at present every day's practice, and no wonder at all." Vol. II., p. 855.

The restriction of operations to particular times of the year, Chelius observes, is no longer considered necessary, although very sultry weather is unfavourable to their issue. Where they can be deferred, a bright light is necessary for their execution, or the patient is liable to rheumatic and gouty affections, they should be delayed until steady weather may be calculated upon.

The elementary acts of every operation consist in Division, Apposition, or Dilatation. Of these, *Division* is the most general and important, and may be effected, 1, by a cut or incision; 2, by a stab or penetration; 3, by tearing asunder; and, 4, by ligature. All instruments employed for *division of soft parts* by *cutting,* must be placed in two classes; those having but a single cutting edge, knives, bistouries, and scalpels, and those "consisting of two cutting edges, connected crosswise in their middle, and terminating in handles," the same forming our old familiar friend a pair of scissors. Then the different kind of knives (and scissors) are described according to the mode in which they are set in the handle or the form of the blade; a mere waste of words which a moment's glance at an instrument-maker's case would render needless. The *straight-edged bistoury* is stated as the only one with which a regular cut can be made, and this is defined as follows:—" A regular cut must have the same depth from its beginning to its end: it must not have any bridges, the angles must not be cut more shallow than the middle, and the edges must not be jagged."

"The mode of holding the knife has an important influence on its use. In this respect, four postures or positions may be distinguished. 1. It is held like a pen, the handle being taken hold of with the thumb and middle-finger near the blade, and the fore-finger laid on its back. Herewith the knife can be used with ease, and employed in every direction. It is especially suitable where small cuts are to be made with great care. 2. The knife is held with the thumb on one, and the middle and ring-finger on the other side of its handle, and the fore-finger laid on the back of its blade, as in holding a violin-bow. 3. The handle is placed on the inside of the ball of the thumb, with the thumb on one side, and the middle, ring, and little finger on the other, whilst the fore-finger is extended upon the back of the blade. 4. The knife is grasped with the whole hand, the thumb on one and the fingers on the other side of the handle. This is only applicable to large or amputating knives.

"Scissors effect the division of parts, like the bistoury, by drawing and pressure; but the pressure is greater, and therefore the scissor edge is generally not so fine as that of the knife; neither are the edges set directly opposite, but lie beside each other, so that ordinarily a cut with scissors is not so clean as that with a bistoury; the part too must also be pressed and squeezed before they are divided. On this account, the use of scissors is by many entirely rejected. The objections, however, to their use may be done away with by the proper fineness of their edge, and by the greater power with which they can be employed. It has been hitherto supposed that the due degree of fineness, like that of a bistoury, cannot be given to scissors without impairing their strength. I, however, possess scissors, made by our clever instrument-maker Görck, which have the perfect edge of a bistoury, and with proper strength." P. 859.

Mr. South has most injudiciously committed himself in a postscript to an unfavourable and somewhat sneering opinion upon the *employment of Ether as a means of preventing pain during operations.* Writing in Feb. 1847, he was not called upon in a systematic work to even notice a remedy only introduced to the profession a few weeks; but, having determined to do so, it is matter of congratulation that his opinion has not been fortified by subsequent experience, and that it will only disfigure his book as a specimen of over-hasty conclusion. Speaking of the propriety of preliminary trials, he says—"With this opinion I fully concur, and I should certainly adopt it, if I made up my mind to try inhalation at all; but upon that point I am not decided, for I have considerable doubt of the propriety of putting a patient into so unnatural a condition as results from inhaling ether, which seems scarcely different from severe intoxication (!)—a state in which no surgeon would be desirous of having a patient who was about to be submitted to a serious operation." Of one thing we are certain, that if some surgeons have not made up their minds upon this matter, most patients have, and that the services of those who, upon mere speculative grounds, decline availing themselves of this means of mitigating dreadful suffering, will very properly be but rarely put into requisition. This is a matter the public must and will decide for itself.

*General Treatment of Lithotomy Patients.*—Mr. South observes that lithotomy cases may prove fatal, however dexterously an operation may have been performed, and he is disposed to refer this to some error in the prior or after-treatment. Upon this subject he offers some very judicious directions, going into minutiæ not to be found in our works on surgery, and for the want of being acquainted with which practitioners may lose patients upon whom the operative procedures have been conducted with skill. He deprecates the placing of patients with stone in the ordinary wards, where they are not quiet enough, and the sisters of which are not habituated to watch various circumstances it is important for the surgeon to be made acquainted with. The following is a brief abstract of the account he furnishes of the practice which has been long followed at St. Thomas's Hospital. The patient is placed in a small ward containing only half-a-dozen beds, and which, after the operation, is kept very private and quiet; and here he remains under the watchful eye of an experienced sister for ten days or a fortnight, to get accustomed to place and attendants. If in tolerable health his diet and habits only demand ordinary attention, occasionally resorting to a hip-bath when his suffering is great, especially after sounding. Rarely is bloodletting or other depleting means practised. The bowels are however to be kept clear; and if the patient have been accustomed to take gin and water to increase the action of the kidneys—a practice very prevalent among such persons—this is to be only discontinued gradually, or even not at all. Temperate weather should if possible be selected, for great heat exercises a very depressing effect on these patients; while, if it be very cold, he may easily become chilled while exposed at intervals during the after-treatment. The day prior to the operation a dose of castor-oil is given, and the diet restricted to rice-pudding and milk, with plenty of barley-water or gruel, especially the former. If the motions are hard and lumpy, an injection of castor-oil and gruel is to be thrown up on the morning, but if not, the gruel alone suffices.

After the operation the patient is put to bed with his legs straight and close together, for the purpose of gently maintaining the edges of the wound in contact, and checking slight hæmorrhage. A napkin is passed around the pelvis just as it is put on an infant, and throughout the treatment this is carefully changed when wet by the urine passing from the wound. In the evening, or next morning, if there be no bleeding, a piece of lint folded on the end of the finger is introduced into the wound, and pressed up the depth of the perineum. It is changed each time the patient wets, and is continued until the healing is accomplished, with the intention of securing this taking place from the bottom, under which plan the formation of fistulous passages is a matter of very rare occurrence. "A handful or two of chamomile flowers thrown into a basin, are sprinkled with spirits of wine, well mixed, so as to be equally moistened, and then put into a thin flannel bag, and having been well heated on a warming-pan, are applied over the belly as hot as the patient can bear, on the evening of the operation-day, if there be no bleeding; and this is continued for a week or ten days." If the wound on the second day is swollen and no urine flows, no lint is introduced, but a bread-and-water poultice is applied until the swelling subsides and the water increases. Cold is not applied, and an opiate is rarely given. The diet for the first two or three days consists of farinaceous food and free libations of barley-water; and a little castor-oil is given on the third day. If there is pain in the belly or sickness, however, these may usually be relieved by giving the oil a little earlier. About the third or fourth day urine usually passes by the urethra as well as through the wound, and in a week or ten days it flows through the natural passage alone, and then the wound is dressed with wax and oil spread on the lint, introduced as before. If the urine does not flow through the wound at first, and the patient becomes irritable and uneasy, the finger should be passed in to remove any obstruction which may be offered by a coagulum. If the patient flag, the resumption of his gin and water may be advisable. He should be kept in bed for some days after the urine has ceased flowing from the wound ; and if the formation of a small sinus retard cicatrization, a piece of lint of corresponding size should be twisted up, dipped in a solution of sulph. cupr., and screwed up to its bottom ; but generally the wax and oil-dressing suffices. In from three to four weeks the cure is completed, the diet having been in the mean time gradually improved, and porter or wine added if deemed necessary.

*Comparison of Lithotomy and Lithotrity.*—This question has lost none of its interest and importance, and from recent discussions at the French Academy, seems as little settled among our brethren across the Channel as heretofore. Lithotrity is there as warmly espoused by the specialists as the preferable operation, as its eligibility is stoutly denied by some of the best surgeons in that country ; and the discovery of Etherization, which seems likely to be a valuable adjuvant in the one operation, and a most questionable means in the other, where the retention of sensibility often affords most useful guidance for the operator, will doubtless render the substitution of lithotrity less common. In our own country, some of our best surgeons, as Brodie, Liston, Key, &c. &c., have shewn that, in many cases, it is the preferable procedure ; and we now have to give an account of the opinion entertained by Chelius. He observes that many of the objections which were tenable at first have lost their validity by reason of the subsequent perfection of instruments and procedures ; but that we have not even yet data upon which any positive opinion can be delivered. The statistics are eminently defective to this end, constructed, as they are, on a faulty basis, while their accuracy and good faith have been warmly and effectually contested at Paris. "If the possible evils which may occur on and after lithotomy and lithotrity be compared, they are found to have a certain degree of equality as to their number and danger; only that in lithotomy the wound especially gives rise to symptoms which in lithotrity are absent, whilst the latter occasions considerable irritation of the bladder, and dangerous symptoms resulting therefrom." After enumerating the various untoward circumstances which may attend either operation, Chelius goes on to say—

"If these various circumstances in lithotomy and lithotrity be compared in regard to their cause; to wit, the wound in lithotomy, and the injury of the bladder in lithotrity, it must be presumed that pain and nervous symptoms may be equally present in both, but their frequent repetition in lithotrity is of importance; that bleeding, wound of the rectum, injury to the peritoneum, which are very much to be dreaded in lithotomy, cannot happen in the modern practice of lithotrity; that infiltration of urine, so frequently fatal after lithotomy, is almost impossible in lithotrity; that phlebitis and peritonitis are observed not unfrequently after lithotomy, but very rarely after lithotrity, which also applies in like manner to the continuance of fistula; that, on the other hand, inflammation of the bladder, inflammation and abscess of the prostate, are more common after lithotrity. Bruising or tearing of the mucous membrane of the bladder, as also breaking of the instruments in the bladder, is at the present time scarcely possible with the improved instruments.

"Further, if lithotrity be considered in reference to the condition of the urinary organs, the age, sex, and constitution of the patient, and the nature of the stone, it follows, that a diseased change and swelling of the prostate, purulent catarrh, great sensibility and contraction of the bladder, render lithotrity quite impossible, or considerably increase its danger. Although lithotrity was formerly considered inapplicable to children, and numerous experiments by Civiale, Amussat, Leroy, and others have proved its practicability in little children; yet, however, the result of lithotomy at this age is so favourable, and the employment of lithotrity so difficult, that lithotomy should undoubtedly be preferred. In advanced age, on the contrary, the results of lithotomy are far more unfavourable than those of lithotrity. (How does the author reconcile this with his statement, that an enlarged prostate contra-indicates lithotrity?) In females, the less difficulty in the introduction of instruments is compensated by the difficulty of keeping the bladder distended; but lithotrity, although lithotomy in woman is much more rarely fatal than in man, has this great advantage, that no incontinence of urine remains after it, an infirmity the importance of which in woman cannot be too seriously thought of. It must finally be remembered that, for very stout persons, who are always the most unfavourable subjects for lithotomy, lithotrity is far less dangerous.

"If now, after the consideration, founded on experience, of the advantages and disadvantages of lithotomy and lithotrity, the particular cases in which the one or the other practice is specially indicated, be reviewed, it follows that lithotrity appears preferable—1, in small stones, or those of no great size; 2, where there are two or several little stones; 3, in stones of moderate size, and when they can be easily broken, and if in all these cases the bladder be healthy, or only in a trivial degree affected. These indications are important, when such cases occur in old persons, in females, or in very stout people. On the other hand, lithotomy is decidedly to be preferred; 1, in childhood; 2, with large and hard, and especially mulberry stones; 3, when there are several large stones; 4, when large stones entirely fill, or are completely locked in by a contracted and unextensible bladder; 5, in diseased prostate, or great affection of the bladder; 6, in very great sensibility of the bladder, so that the patient can bear neither its distension, nor the motion of the instruments; 7, with stones of which the nucleus, as for example, when it is a bullet or the like, cannot be destroyed by the lithotriptor. It is also not to be overlooked that, in the general employment of lithotrity, the patient should be subjected to it early, by which its results are more certain, and its use will become more easy and general. On the other hand, however, it must not be unnoticed, that under directly the same circumstances which are favourable for crushing, does cutting for the stone, if performed with ability, lose much of its danger. Vol. II., p. 623—5.

After detailing the opinions entertained by some of our best surgeons favorable to lithotrity, Mr. South goes on to say:

"I do not propose to offer any opinion of my own as to the preference which should be awarded to lithotrity or lithotomy, as I have had little practical experience in regard to the former, and am therefore not qualified to give one. But I may be permitted to say that the results of the practice of lithotomy, both with gorget and knife, and by various operators on patients of all ages and under various circumstances, during the course of a long series of years at our hospital, have been so favourable, as to afford little cause for making it give place to lithotrity. I think it is proved that lithotomy, when properly conducted, is not the dangerous operation it is too commonly held to be; and it is no trifling advantage it possesses, that the patient is relieved at once, with a few minutes' suffering, sharp indeed it must be acknowleged to be, instead of being subjected to several operations, which the more frequent in their repetition, become, as generally admitted, greater in severity, and occasionally leave the necessity for resorting to the cure by lithotomy. I may here also add the testimony of some patients who have undergone both operations, that the suffering during lithotomy was less than in lithotrity, and that knowing both, they would, if needful, prefer undergoing the former. It is well, however, that we have the opportunity of employing lithotrity in cases where patients are too fearful to submit to the knife; but I am by no means sure that, under all circumstances, lithotomy is not at least as free from danger as lithotrity, and certainly more speedy as regards cure." P. 629.

A tolerably strong opinion this for one who professes his inability to offer any; and one which we will venture to say will not receive general assent. Although the operations at St. Thomas's may have proved as successful as stated, and we have heard of individual operators, both in this and other countries boasting of their never, or hardly ever, losing a case; yet the untoward events which attend lithotomy are of too frequent occurrence to be disposed of in this way. So too the few minutes sharp suffering, here so quietly spoken of, is too often prolonged, and that sometimes under the best hands, to a far more protracted period of intense agony. Mr. South limits his preference to the operation to examples of it "properly conducted;" but it is but just to mete out a similar restriction to the rival proceedure, which most certainly could never have embraced the cases he alludes to. Nor do we see how this is ever to be the case if suitable instruction be not given to the students of our hospitals in the use of lithotripic instruments—the more dashing results of lithotomy even yet almost exclusively occupying their attention. It seems to us certain enough that if we were restricted to the one operation, lithotomy would necessarily be the one preferred, as embracing many cases lithotrity is not competent to cope with: but, enabled as we now are to adapt each procedure to appropriate cases, it is surely but reasonable to select the least painful and least formidable one for such as satisfactorily admit of its being put into force. The grounds of the selection may yet occasion some difference of opinion; and we certainly cannot agree with Chelius, who prefers lithotrity in the aged, or with other surgeons who employ it in the cases of children. Perhaps the greatest benefit which lithotrity has conferred is the inducement it offers for patients to submit and surgeons to undertake the curative treatment of stone-cases at a far earlier stage of the case than was formerly the practice—thereby preventing much of that secondary disease of the urinary organs which rendered the results of operations more doubtful and the recurrence of the disease more probable.

Mr. South furnishes us with a table of the 295 operations performed at St. Thomas's Hospital from 1800 to 1846; and a second table containing the dates and results of most of those which have occurred since 1822: the table being compiled from various sources and yet confessedly very imperfect, owing to the disgraceful negligence which has prevailed, until quite a recent period, at this and our other hospitals in the keeping minute and accurate records of the cases treated within their walls. It is singular enough to find one of the surgeons of that hospital now acknowledging the assistance he has derived from the "*Lancet,*" in rescuing several of these from oblivion. With all its early faults, who can doubt the great services that publication has rendered to the profession by laying open the rich veins of information buried in those establishments, or only existing therein for the advantage of the favoured few? In the 144 cases of this latter table we find 124 cures and 15 deaths recorded, and five cases in which the results are not reported; but, to make up the number of operations which have taken place in the hospital since 1822 (the date of this last table), viz. 159, we have to add another 15 cases of which no record whatever seems to have been kept. From a so-called statistical table like this, it is obvious that no safe conclusion can be drawn, and we cannot therefore agree with Mr. South, that meagre as the reports are, "they are highly important, as shewing that the lateral operation is neither so dangerous, nor so much to be dreaded, if the after-treatment be well attended to." A statistical table, which gives us no account whatever of the results of 20 out of 159 operations is obviously useless, and may become, if relied upon, positively mischievous. As age is an important item in the estimate of this operation, we may mention that, of the 125 cases in which the age is recorded, 58 operations occurred in children 10 years old and younger; 26 in young persons from 10 to 20 years of age; 17 in persons aged from 20 to 50; and 24 in persons aged more than 50 years.

*Radical Cure of Hydrocele.*—Chelius acknowledges a preference of incision of the tunica vaginalis to its injection, which will certainly meet with no assent from surgeons in this country. He says—

"It is not advisable to produce upon the testicle any irritation like that in the vaginal tunic. By incision, all the complications can be most distinctly made out, at the same time any existing intestinal rupture can be properly treated, the inflammation be more properly excited, and effect a more safe cure. The bleeding which occurs in this operation is easily stanched; the severe symptoms occurring after it are most commonly the result of bad practice. After the cut it is in most cases necessary to insert a half-unravelled piece of linen between the wounded edges of the vaginal tunic. Injections operate uncertainly, as the irritability of the individual cannot be previously determined. They act as violently on the testicle as on the scrotum; if a part of the injection be poured into the the cellular tissue, which is possible, even with the greatest care and attention, very dangerous symptoms may arise therefrom; in a diseased state of the testicle, which cannot always be decidedly made out, injections are necessarily hurtful. The superiority of injections, to wit, that by their use the cure follows more quickly, and that the patient does not need to be kept quiet so long, is of no value, as even after injection the cure is often longer protracted than after incision, and with the latter keeping so long quiet is not necessary." Vol. II., p. 506.

Few surgeons, we imagine, who have witnessed the usual successfulness of so simple an operation as the injection of a hydrocele, will be disposed to adopt a procedure which requires the opening up the whole length of the tunica vaginalis, "care being taken that the testicle do not protrude, and if it should, it must be gently returned," and stuffing the wound with a "half-unravelled piece of linen." Mr. South speaks highly of the use of *Iodine*, so strongly recommended by the Indian practitioners and by Velpeau as the material for injection.

"The injection of tincture of iodine diluted with water, I am convinced, by repeatedly practising it for some years, is the most effectual and least painful to the patient. Velpeau has the credit of introducing the practice, but I am informed by medical friends who have been in India, it has been long practised there (this is acknowledged by Velpeau and every one else), and, if my recollection be not treacherous, without drawing the injection off again. And this mode I have adopted, making use of a very fine trocar and canula, drawing off the water, and injecting an ounce of fluid containing ʒij. Tr., and ʒvj. W., and then immediately withdrawing the canula, to which the wound always clings very tightly, as the solution is very astringent. The patient most commonly suffers no pain, or at least a very trifling degree; and although on the following day the scrotum is a little reddened and rather firm, yet the patient is not thereby prevented from moving about with ease and comfort to himself. Indeed, I am informed that as soon as the injection is made the person walks away, and requires no farther attention. The scrotum, according to my own observation, increases a little, and becomes rather more solid for three or four days, and then begins to subside, and in the course of a fortnight the cure is completed without confining the patient more than two or three days, rather as precautionary, than that I really believe it necessary. I have employed this treatment both in large and small hydroceles, merely injecting the quantity mentioned or a little less; and never either shaking the scrotum about or distending the cyst by repeated injection, and afterwards drawing it off, as Velpeau practises and recommends. I am quite sure whoever enploys the iodine injection as I have mentioned, will not treat a hydrocele for the radical cure by any other means." P. 504.

*Question of Removing a Scirrhous Breast.*—We believe the experience of the best-informed surgeons is increasingly adverse to the undertaking this operation. Formerly it was believed that the unsuccessful results depended upon its being too long delayed; but more accurate and extended observation prove that this is only one of the elements necessary to be taken into consideration. Mr. South quotes the statistics of Leroy d'Etiolles (*Bulletin de l'Academie,* vol. ix.), from which it appears that, taking the mean of the results of 300 operations in men, the duration of life was found to have been 3 years 9 months between the appearance of the disease and the operation, and 1 year 5 months after the operation. In 412 operations on women, it was 3 years 6 months before, 2 years 6 months after the operation. Of 801 cases 117 were operated upon in less than a year after the appearance of the disease, and in 61 the disease was known to have returned. Great differences prevailed according to the tissue affected. Of 633 men suffering from cancer 165 were attacked in the *lip;* while of 2148 women but only 54 were so affected—the difference probably depending on the use of the pipe, which may also serve to explain the greater proportion of relapses which occur in women than men. In 22 operations upon women there were 7 returns; in 114 operations upon men 15. Cancer of the *tongue* is also of more frequent occurrence in man; but the termination in either sex is equally unfavourable. Of 633 cancers in men 18 were seated in the tongue; and of 2148 in women 2 only attacked that organ. Of 9 operations 6 died of relapse, a year not having yet elapsed since their performance in the other cases. Of 277 operations on the *Breast,* 73 were performed within less than two years, and M. Leroy could not therefore furnish the results. Of the remaining 204, there died 22 in the year after the operation, and 87 had a return, making the whole relapses 109, or more than a half.

Chelius sanctions the operation in cases wherein few would undertake it in this country. "Where the scirrhus is already in the state of concealed cancer, the nipple much drawn in, the skin less free and moveable, the general health affected, menstruation irregular or entirely ceased, the result of the operation is indeed doubly doubtful. It is, however, the only remedy to prevent certain breaking. If the scirrhus be already ulcerated, if it be immoveably connected with all the pectoral muscles, if there be also hardening of other organs, no cure is indeed to be expected from the operation. It may, however, in so far, in such a case, be considered as a palliative, as the patient is at least free from the great inconvenience attendant upon the destruction of a scirrhous tumour by ulceration. I have not noticed a quicker progress of the disease after the operation, but, on the contrary, considerable relief for a long while." This would seem to justify its performance at any stage; but a few lines farther on we find a qualification of the advice. "It must not be overlooked, in deciding upon the removal of a scirrhous breast, that in cases where cancer has been very slowly developed and accompanied with no great pain, that after the operation the ulceration again proceeds even quickly, and thus the operation only hastens the fatal result." That it does so likewise by urging into activity dormant cancer of the internal organs numerous examples prove. After citing the experience of A. Cooper and Brodie in proof of the great liability to quick relapse and speedy death after operation, and alluding to two or three cases in which such relapse has been delayed for many years, Mr. South continues:

"Notwithstanding these few favourable instances, surgeons should be cautious in urging a patient to submit to an operation for a scirrhous tumour, and still less, when it has become a cancerous sore, and the neighbouring glands have in either case become affected. He cannot promise a cure by the operation; nor can he even say that the patient's condition will not be made worse. I have often heard it stated that though the operation will not cure, it will put off the evil day, and retard the ulcerative process: but this I do not believe, for I have known many instances to the contrary. The only thing an operation can do is temporary palliation, if the patient be subject to severe shooting, stabbing pain, which is not indeed very commonly the case, unless the disease be worried by local attempts to cure. The practitioner ought, when consulted under these circumstances, to break to the patient cautiously the nature of her complaint; should inform her that all which can be done by operation is at least merely palliative; and should leave her to decide upon whether she will yield herself to the operation, knowing the risk and slender hope connected with it; rather than urge her to an operation which is without doubt, as regards scirrhous swellings, the most unsatisfactory in the whole course of surgical practice." Vol. II., p. 797.

*Application of Blisters.*—Mr. South supplies some useful hints under this head. He observes that the most cleanly and efficient mode of forming a blister is to sop a fold or two of lint in the acetum cantharidis and apply it to the part for a few hours. When the skin is irritable and thin, it suffices to freely apply it by means of a camel's hair pencil. When the blistering plaister is, however, employed, a piece of tissue paper should intervene between it and the skin, which, if the blister be closely applied, will not impede its action, while it will prevent small portions of plaister adhering to the skin or cutis and causing much unnecessary torment. The length of time the blister is to be kept on is too often left to the nurse's decision. It should not be retained on longer than the skin becomes fairly raised, which is usually the case in from six to eight hours. (And we may observe that the blister acts much more rapidly if its surface be previously well moistened with Spt. of Turpentine). In *children* it should be taken off as soon as a bright redness of the skin is induced, which is generally in two or three hours. "Indeed with children I am by no means sure that, in most cases, a mustard poultice is not preferable to the application of a blister. It should be made with mustard (not such as has been long in the house) and warm water, rather thinner than if for the table, as if made stiff it is much less active. It should then be spread about a quarter of an inch thick on fine muslin, and another layer of muslin being put upon it, applied to the part, and kept on ten, fifteen, or twenty minutes, according to the redness and pain. In some persons it will even blister. When removed, the skin should be carefully sponged clean with warm water, otherwise the irritation, which is very great, will continue. In the few persons whose skin is blistered with difficulty, it is best to apply previously a mustard poultice till the skin becomes reddened and painful." Mr. South disapproves of open blisters, as giving only unnecessary pain, and in cases where these seem indicated prefers a rapid succession of small blisters about the size of half-a-crown "Another very excellent and very gentle mode of blistering is with *croton-oil,* ten or twelve drops of which should be gently rubbed over the surface with the finger, protected in a piece of oiled silk, for two or three following nights. Usually slight stinging is felt, accompanied with puffiness of the part on the second or third day, and this is followed by a crop of small vesicles, which speedily mature, in a day or two after dry up and fresh cuticle is formed. It is one of the best modes of blistering (?), if not required to be speedy."

*Amputation.*—Mr. South gives some useful directions for the use of the *Saw,* which, if we may judge from the bungling manner we have seen this instrument employed by hands not otherwise inexpert, are not uncalled for.

"A good saw should have its teeth well set off, as the carpenter's expression is, that it may neither clog or hang in its track; and it should have, proportionally to its size, a heavy back, which renders its steadying more easy, and affords all the weight the saw requires to be loaded with. The too frequent mode of using it, is to drop its end, whilst the handle is raised, so that when moved it works obliquely; the operator, at the same time, throwing as much of his own weight as he can conveniently spare upon the handle, as if with the intention of forcing the blade of the saw at one or two strokes through the bone, and then driving it downwards and upwards, as violently and as quickly as he can, and often using about as much of the toothed edge as a young violinist does of his fiddle-bow. The consequence is, that the saw works badly, is continually jumping out of its track, makes another, and finishes by splintering the bone, and often cutting through it below where it was purposed. To use a saw properly, it should always, where possible, be held and worked horizontally, moving it forwards and backwards without any pressure of the hand, but allowing merely its own weight to keep it on the appointed place; and as it is moved forwards, even its own weight should be lessened, by slightly supporting instead of pressing down the saw. After drawing the toothed edge at first backwards, and then moving it forwards lightly on the bone, till a shallow track is made, it may be moved freely, so that at least two-thirds, or even more, of the saw shall act. The strokes should not be quick but long; and if so made, four or six of them will cut through the thigh or shin bone more quickly and more cleanly than twice as many short, hurried strokes, and without any risk of splintering the bone, or slipping from the part chosen to saw through." Vol. II. p. 893.

*Results of Amputation.*—The result of Mr. Philipps' statistical enquiry is, that the mortality in France, Germany, America and England put together, is $23\frac{7}{16}$ per cent. In 276 cases of amputation which have occurred at the Glasgow Infirmary, Dr. Laurie states there have been 176 recoveries and 100 deaths. Potter states that among 66, performed at University College Hospital in 1835-41, there were but 10 deaths, three of these occurring in instances of immediate amputation after accidents. Mr. South has performed 54 amputations at St. Thomas's in 1835-40, and among these 41 patients lived and 13 died, which he says is the general proportion at St. Thomas's. "Putting these together with the cases at University College Hospital, it must be evident that the mortality is a long way below 50 to 75 per cent., which has been stated by some surgical writers as the ordinary average of amputations. It will be observed, also, that the largest mortality is among the cases operated on for accidents, and on the lower extremities. In 7 amputations through the thigh I lost 6; and of 9 through the leg 3 died. Whilst of 6 primary and 1 secondary amputations in the upper extremity, not a single case was lost. This excess of mortality in operations after accidents is to be ascribed, when the patients die early, to the conjoined shock of the accident and operation. Besides which, the persons admitted into hospitals for such injuries are commonly free livers with broken-down constitutions, the like of whom are not unfrequently destroyed by the results of trivial accidents, which run either into erysipelas, or diffuse inflammation and gangrene."

*Circumcision in Phymosis.*—The mode of performing circumcision among the Jews is thus described. The child being held across the thighs of a man who is seated, the circumcisor grasps the prepuce with the thumb and fore-finger of his left hand, draws it forwards, and inserts it in the cleft of an instrument similar to a silver spatula. The penis is held upright, and the prepuce cut close off to the spatula with a single stroke of a button-ended knife. The circumcisor then, as quickly as possible, seizes the inner fold of the prepuce with his thumb-nails, which have been specially cut for the purpose, and tears it up to the corona glandis. He then spirts some water from his mouth over the wound, takes the penis in his mouth, and sucks the blood out of it a few times. A strip of fine linen is now wound round the corona and the cut surfaces, as a dressing, and the penis laid upon the pubes, in a ring to prevent its being touched.

Mr. South observes, in respect to this ancient and effectual procedure:

"Many years ago I was present at a Jewish circumcision, and was so much struck with its facility and appropriateness to the purpose, that I have ever since performed the operation in the same manner, except that, instead of inserting the prepuce in the cleft spatula, I merely grasp it with a pair of dressing forceps, as close as possible to the glans, and then cut it off before them. The tearing up the inner part of the prepuce to the corona is a very important part of the operation, and far preferable to its division to that extent with the knife, as whilst the inflammation is subsiding, the cut edges, especially near to the angle of the wound, are prone to adhere together by quick union; and even if this do not extend far it causes a girthing of the glans, which is inconvenient and often requires a second division to complete the cure. By tearing the inner skin, which should always be torn completely behind the corona, or the operation will be useless, the edges of the wound become sloughy, and disposition to quick union is prevented. From having repeatedly performed circumcision in this way I am sure it is the best mode. And I may add that, as regards circumcision or slitting up of the prepuce, the former is in every case much to be preferred. I never, however, put in any stitches, as they are not merely superfluous, but add to the necessary inflammation without sufficient reason." Vol. II., p. 345.

*Reduction of Paraphymosis.*—We have so frequently seen the operation of paraphymosis—so painful in its performance and oftentimes followed by such tedious ulceration—harshly and unnecessarily resorted to that we are desirous of transcribing Mr. South's excellent remarks upon its reduction, although they are rather long.

"The reduction of paraphymosis is often exceedingly difficult, and always excessively painful, so that frequently a strong-minded person will scream like a child from the pain. I have, however, scarcely ever known it fail, and hardly remember it needful to perform any operation with the knife. It requ res, however, great patience and perseverance, often for the space of half-an-hour, at the very least, and I have often succeeded when the prepuce had been everted six or eight days, and it might have been supposed that the adhesive inflammation would have prevented the replacement of the skin. Although the diminution of the bulk of the glans, by pressing the blood as completely as possible out of it, is a very important part of the proceeding, yet squeezing out the fluid effused in the prepuce is no less so; and unless both be done, there is great hindrance to the replacement of the skin. I therefore always first squeeze gently but steadily, for a few minutes, the prepuce, till it become somewhat flaccid, and then firmly press the whole glans with the thumbs of both hands, whilst the two forefingers grasp the penis behind the prepuce, like a collar, and draw it forwards, whilst the thumbs empty and thrust back the glans. Or if I do not so succeed, I grasp the whole penis with my left hand, making the thumb and forefinger a collar behind the inverted prepuce, which thus rests against it, whilst, with the thumb and fingers of the other hand, the glans is emptied, and thrust within the constricted ring, by pushing first one part and then another of the corona glandis till it gets beneath the constricting band; and this done, the rest soon follows. Immediately that the least bit of the corona has been thus got in, that next it must be poked in (no better expression than this can be used) with the finger end, and so the next, until the greater part has been thus returned, and the reduction is speedily completed. It must, however, be remembered, that directly the return has commenced, the poking must be continued without intermission, as otherwise the whole proceeding will have to be repeated, as, on the least cessation, the glans again fills, and protrudes. If the everted prepuce do not relax by pressure, the constriction being so great that the effused serum cannot be dispersed easily upon the body of the penis, it will be found very convenient to make a few punctures through the skin, by which the squeezing presses out the fluid, and then the prepuce is rendered flaccid. It is always desirous to try this mode of proceeding, even if a part of the prepuce should have become gangrenous, as this is often merely superficial, and the replacement puts a stop to its progress. After the reduction, it is well for some hours to wrap the penis up in linen, and to keep it constantly wet with cold water, for the purpose of preventing the disposition to erection, and reprotrusion of the glans, but after that time, a warm poultice will be most agreeable to the patient's feelings, and most favourable to the dispersion of the inflammation. The soreness, however, will commonly continue for many days, proportionate to the severity of the constriction and its duration. No attempt at retraction of the prepuce, to see what is going on inside, should be made for several days, or the mischief will probably recur." Vol. II., p. 350.

*Curvatures.*—Chelius takes a comprehensive general view of these before entering upon a description of the different varieties. As the erect position of the body and its various organs depends upon the *equal antagonizing operation of the muscles,* and on the *firmness of the bones,* whatever interferes with the muscular antagonism or operates changes in the osseous structure, is competent to induce curvature. Muscular antagonism may be disturbed either by the preponderating activity of certain muscles, or by the mere normal action of others operating against an enfeebled condition of their antagonists. This last condition may result from palsy, wounds, debility of the muscles, their continued rest, spasm, continued exertion in certain positions, especially in children, or from diseased changes in muscle produced by gout, rheumatism, ulceration, ossification, &c. The activity of the flexors, especially in the fœtus, exceeding that of the extensors, the greater number of congenital and original curvatures occur in the course of the former. Muscles which produce the curvature always undergo more or less contraction or shortening, so as to be capable of little or even of no extension; and if this condition persist they lose their fulness, become thin or even cord-like, and are at last converted into a fibro-cellular or fatty mass. These changes are always the same whatever cause may have produced the contraction of the muscles, and are due to the *continued rest* of these parts—the tissue becoming atrophied, just as occurs in other unused parts.

"The rest of a muscle, when its contraction has once taken place, is therefore continual, because all voluntary motions which the patient attempts with the curved part, can occur only in such one way and direction, that thereby no outstretching and extension, but only a greater shortening of the contracted muscles, can be effected. A close observation of the motions in curvatures, especially in the feet, shows this remarkably. It is clear, that under such circumstances, the nervous influence and nourishment must be diminished in the muscles, and the diminution of the nervous influence may increase up to actual palsy, although the contraction of the muscle continue. In this way also is explained the reason why in palsy, which originates from the brain, and loss or diminution of the voluntary influence depending on the muscles, the muscles are contracted, whilst in palsy, proceeding from the spinal marrow, they are lax and atonic. Spasm, produced by topical causes in the muscle itself, or by reflected activity of the spinal marrow, may be the first origin of contraction of the muscles, and of the curvature thereon depending; but the continued contraction of the muscle is not to be considered as a consequence of *continual* spasm, but of the sustained rest of the muscle, and its diminished voluntary action. The same is observed in inflammation, and in all painful affections when certain muscles are kept in a continued quiet state. The most direct proof of this opinion is given by the bearings of the limb, if its natural direction be restored, in which case the recapability of motion, and the voluntary influence again gradually returns, and in the same measure, the nutrition of the muscles is increased, and their bulk enlarged, as I have especially observed, after the cure of curvature by cutting tendons." Vol. II., p. 150.

The normal condition of the bones may be disturbed by rickets, osteomalacy, scrofula, inflammation, &c.; when the softened bones yield to the action of the muscles, or the weight of the body.

The *prognosis* of curvatures depends upon their extent and duration and the manageable character of their original causes—being the more favorable the younger the subject and the less the curve. In old and bad cases our agency will be limited to the prevention of an aggravation of the malady; while, when organic changes of the bones and joints have occurred, the case becomes incurable. "Curvatures resulting from muscular contraction usually admit of a more favourable prognosis than those which proceed from diminished connexion of the bones. But if the muscles have become so wasted by long-continued curvature that their lengthening can be of no use, which is, however, difficult to determine, they are incurable."

The *cure* of curvature depends on the removal of the causes and the restoration of the natural direction to the parts. In a diseased condition of the bones no benefit can result from a mere mechanical treatment, unless such condition be rendered amenable to the therapeutical procedure adapted for it. When dependent upon a disturbed equilibrium of the muscular activity the cause of this must be sought for. The frictions usually recommended are chiefly useful from the motion and extension of the contracted parts which their employment renders necessary. On account of the diminished nervous influences in the parts, counter-irritation may act beneficially by quickening and increasing the vitality, especially in actual palsy. Kneading, rubbing, and stretching the muscles, and suitable gymnastic exercises will, with due attention to the general health, remove the slighter curvatures, but where these are considerable, suitable machinery and apparatus are requisite.

"If with long-continued curvature from shortening of the muscles, such change of their tissue have been produced, that by the treatment proposed it can be removed either with extreme difficulty, or not at all, the subcutaneous cutting through the shortened muscles, or their tendons and aponeuroses (*myotomia, tenotomia*) if possible, is the most proper remedy. Between the two ends of the divided tendon which retract, the upper more strongly than the lower, blood is effused, which coagulates and unites with the whole internal surface of the wound, and especially with the ends of the tendon. Exudations of plastic lymph soon occur, particularly from the ends of the tendon, presenting whitish thread-like streaks, running from one to the other, and gradually form a mass resembling fibrous tissue, which is capable of due extension, and sufficiently strong to answer the function of the muscles. This operation is therefore especially indicated under the above-mentioned conditions, if there do not at the same time exist such considerable changes in the bones, and from the long continuance of the disease, such a degree of wasting in the muscles and the whole limb, that by the mere lengthening of the muscles, the restoration of their natural position cannot be effected, which, however, it is often difficult previously to determine, and when the causes giving rise to the contraction, gout for instance, still exist. The various objections made to this operation, the repeated shortening of the tendons by the gradual contraction of the newly-formed intersubstance, as observed in every scar, as well as the injury to the natural direction and motions of the part from excessive activity of the antagonizing muscles, are without foundation, and contradicted by the large experience of modern times. The pain and wound are usually slight, and no particular symptoms occur. If in rare cases such be observed, as violent inflammation, with destruction of the cellular tissue, exfoliation of tendons, and so on, they must be ascribed rather to the peculiar relations of the constitution of the patient, or to the proceedings in the operation and the after-treatment, than to the operation itself. The straightening of the part, and the stretching of the tendons and proper apparatus, is most properly commenced some days after they have been cut through, when the external wound is healed, to which time a light bandage covering the parts keeps it in a proper position. The employment of extension immediately after the division is improper, as thereby the two ends of the tendon are too far separated, and bad symptoms may be brought on. Too late use of extension, when the intermediate substance has attained firmness, renders the lengthening difficult, and even impossible."—P. 151.

In taking leave of Mr. South's labours, we must join in the regret we have frequently heard expressed, that, with so much industry and so large an accumulation of experience at command, he did not rather produce an original work than undertake this translation. We cannot compliment him upon the elegance of his rendering, many of the sentences being very obscure, and others ungrammatical, as our readers may perceive by our extracts. As we before stated, however, the work will be found very useful to both student and practitioner; and we heartily wish the spirited speculation of its production the success it deserves.

